# FGF/FGFR Pathways in Multiple Sclerosis and in Its Disease Models

**DOI:** 10.3390/cells10040884

**Published:** 2021-04-13

**Authors:** Ranjithkumar Rajendran, Gregor Böttiger, Christine Stadelmann, Srikanth Karnati, Martin Berghoff

**Affiliations:** 1 Experimental Neurology, Department of Neurology, University of Giessen, Klinikstrasse 33, 35385 Giessen, Germany; Ranjithkumar.Rajendran@neuro.med.uni-giessen.de (R.R.); gregor.w.boettiger@med.uni-giessen.de (G.B.); 2 Institute of Neuropathology, University Medical Center Göttingen, Robert-Koch-Strasse 40, 37075 Göttingen, Germany; cstadelmann@med.uni-goettingen.de; 3 Institute of Anatomy and Cell Biology, University of Würzburg, Koellikerstrasse 6, 97080 Würzburg, Germany; srikanth.karnati@uni-wuerzburg.de

**Keywords:** FGF, FGFR, multiple sclerosis, EAE, ERK, Akt, BDNF, LINGO-1, SEMA3A

## Abstract

Multiple sclerosis (MS) is a chronic inflammatory and neurodegenerative disease of the central nervous system (CNS) affecting more than two million people worldwide. In MS, oligodendrocytes and myelin sheaths are destroyed by autoimmune-mediated inflammation, while remyelination is impaired. Recent investigations of post-mortem tissue suggest that Fibroblast growth factor (FGF) signaling may regulate inflammation and myelination in MS. FGF2 expression seems to correlate positively with macrophages/microglia and negatively with myelination; FGF1 was suggested to promote remyelination. In myelin oligodendrocyte glycoprotein (MOG)_35–55_-induced experimental autoimmune encephalomyelitis (EAE), systemic deletion of FGF2 suggested that FGF2 may promote remyelination. Specific deletion of FGF receptors (FGFRs) in oligodendrocytes in this EAE model resulted in a decrease of lymphocyte and macrophage/microglia infiltration as well as myelin and axon degeneration. These effects were mediated by ERK/Akt phosphorylation, a brain-derived neurotrophic factor, and downregulation of inhibitors of remyelination. In the first part of this review, the most important pharmacotherapeutic principles for MS will be illustrated, and then we will review recent advances made on FGF signaling in MS. Thus, we will suggest application of FGFR inhibitors, which are currently used in Phase II and III cancer trials, as a therapeutic option to reduce inflammation and induce remyelination in EAE and eventually MS.

## 1. Multiple Sclerosis Is a Chronic Disease of the Central Nervous System

Multiple sclerosis is a chronic inflammatory and neurodegenerative disease of the CNS. Acute and chronic lesions in the CNS are differentiated. In acute lesions, active demyelination and inflammation are present, whereas in chronic lesions, mainly loss of myelin and gliosis is found [1,2]. Lesions are present throughout the CNS, including the spinal cord, brain stem, and periventricular areas of the cerebrum. In addition, brain tissue adjacent to the subarachnoid space is especially vulnerable to demyelination. Further, mild meningeal inflammation with lymphocytes, plasma cells, and macrophages is common in MS pathology [3]. MS affects more than two million people worldwide [4]. In the majority of patients, the disease begins with a single episode (or relapse) of a neurological deficit involving the optic nerve, brainstem, or spinal cord. The most common condition is called relapsing remitting multiple sclerosis (RRMS), which affects individuals mostly early in their adult life (mean age at onset of approximately 30 years), however, around 20% of patients have late-onset RRMS with an onset of more than 40 years [5]. The male population with late-onset RRMS reached severe disability faster than those with young RRMS [5]. In addition, polypharmacy, the condition of using multiple medications, was more common in older RRMS patients with high BMI [6]. RRMS occurs more often in females than in males (female/male ratio of 2.7:1) [7]. Among patients with relapsing onset, 62% develop moderate, 29% severe disability, and almost 40% of patients with relapsing onset develop a secondary progressive disease course [7]. Primary progressive MS is a rare disease type affecting 10–15% of patients. The 2017 revision of the McDonald criteria, mainly based on clinical and MRI findings, is the current diagnostic classification system for MS [8]. Patient-reported outcomes (PROs) are increasingly used in clinical practice to improve patient-centered care for MS [9]. Environmental (e.g., vitamin D deficiency, diet, obesity in early life, cigarette smoking, Epstein Barr Virus (EBV) infection as a young adult), genetic, and epigenetic factors were suggested to contribute to the etiology of MS [10]. Health-related quality of life is reduced in patients with higher disability [11]. Comorbidities are frequent in MS, and they can affect the outcome [12]. Today, most patients with RRMS are treated with disease modifying treatments (DMT) such as fumarates, the adhesion molecule blocker natalizumab, or sphingosine 1-phosphate (S1P) immune cell migration inhibitors. The costs of MS from the societal perspective are high. In Germany, the disease causes significant disability, and dependent direct (healthcare) and indirect costs (absence from work, early retirement) of up to 60,000 EUR per patient in a year [13].

## 2. Inflammatory Destruction of Oligodendrocytes and Myelin Sheaths

Myelin sheaths are important for the maintenance and protection of axons [14]. In MS, degeneration of myelin is a result of inflammatory destruction of oligodendrocytes and myelin sheaths. MS pathology is associated with the development of large, demyelinated plaques, oligodendrocyte destruction, and axonal degeneration in the CNS [15]. The adaptive immune system is considered to contribute significantly to the pathogenesis of MS. T cells and B cells are selectively recruited by target antigens expressed in the CNS [16]. It is still unclear how immune responses against CNS structures are initiated in MS. The initial step could occur in the CNS, where CNS antigens could be released to the periphery, initiating a subsequent autoimmune response against structures in the CNS. Alternatively, the initial step could take place in the periphery, with the subsequent aberrant immune response targeting the CNS. Both models would include immune responses in peripheral lymphoid tissues and the migration of peripheral lymphocytes over the blood-brain barrier into the CNS. In MS, the endothelium of the blood-brain barrier shows increased expression of adhesion molecules, proinflammatory cytokines, and chemokines combined with reduced expression of junctional molecules, allowing for increased recruitment of circulating leukocytes [17]. Further, the innate immune system plays an important role in the initiation and progression of MS. Microglial activation in the CNS contributes to the pathology by secretion of proinflammatory cytokines, chemokines, and free radicals [8]. Apart from this autoimmune-mediated inflammatory destruction of CNS structures, oxidative and endoplasmic reticulum stress may cause damage to oligodendrocytes [18,19]. MS lesions are distinguished by their demyelinating activity and immune cells [20]: in active demyelinating lesions, myelin-phagocytosing macrophages within lesions or at lesion edges are found, and in chronic inactive lesions demyelination has stopped, though T cells may still be present in perivascular regions. Macrophages phagocytosing myelin are considered to be activated by CD4^+^ T lymphocytes in the periphery. In addition, apoptosis of oligodendrocytes can be directly induced by activated CD8^+^ T lymphocytes. MS pathology includes the loss of oligodendrocyte subpopulations [21]. It was reported that prior to ongoing phagocytosis, certain acute lesions are present without lymphocyte infiltration but already show loss of oligodendrocytes, arguing for an initiation in oligodendrocytes themselves. However, in animal models, induction of oligodendrocyte cell death alone does not provoke immune responses, as seen in MS. Overall, current understanding of the pathogenesis of oligodendrocyte loss is based on secondary to activation of T lymphocytes and monocytes against myelin antigens [22]. The ability of macrophages to remove myelin debris after destruction of the sheaths is an integral part of the recovery process; successful clearance of myelin presumably precedes remyelination [23,24,25,26]. The destruction of myelin sheaths is associated with axon degeneration, which is the main underlying mechanism of permanent disability in patients [8]. However, neuronal damage can precede myelin damage or develop without white matter pathology. Cases where grey matter damage is independent of white matter lesions underline this. Models of mitochondrial injury may explain how neurons are damaged while ensheathment is uninjured. Both mechanisms—damage to sheaths and trophic insufficiency—may be independently involved [27]. Concordantly, axon degeneration takes place in early lesions and later stages of MS [28,29]. The reason for the ongoing destruction of axons is unclear; lack of axonal support caused by degenerated myelin may be one reason for it [30]. Another mechanism involved may be glutamate induced excitotoxicity. Activated microglia induce loss of amino acid transporters and disrupt glutamate metabolism [31]. Taken together, it remains to be resolved if retrograde destruction due to demyelination, prolonged microglial activation, or loss of trophic support is the pivotal mechanism for neurodegeneration [27]. Following demyelination, however, oligodendrocytes undergo a complex program of proliferation, migration, differentiation, and myelination to produce myelin sheaths [32]. Oligodendrocyte lineage cells differentiation can be subdivided into three stages based on marker protein expression, i) oligodendrocyte progenitor cells (OPCs), ii) premyelinating oligodendrocytes, and iii) myelinating oligodendrocytes [33]. OPC depend on factors stimulating their activation, recruitment, and differentiation [34]. In response to injury, OPC undergo a switch from a quiescent to a regenerative state. Microglia and astrocytes activated upon injury are the major source for mitogens and pro-migratory factors for OPC. In MS, remyelination often fails, which may be explained by reduced recruitment of OPC to demyelinated areas, and impaired differentiation of OPC into myelinating oligodendrocytes [35]. Positive regulators of OPC differentiation in MS are found in acute areas of inflammation and in early stages of lesion formation [36], but they are absent in a chronic inflammatory environment [37], where inhibitors of oligodendrocyte differentiation, such as TGF-β (Transforming growth factor beta) and semaphorin 3A (SEMA3A), are present [34]. The difference in efficiency of myelin regeneration between acute and chronic MS lesions suggests that there is a window of opportunity for remyelination early in the disease. Therefore, strategies to induce remyelination may include the removal or neutralization of the inhibition of OPC differentiation at early stages of MS or the alteration of the inflammatory environment. Leucine rich repeat and immunoglobin-like domain-containing protein 1 (LINGO-1) are key inhibitors of oligodendrocyte differentiation [38]. Although successful in a MS disease model [39], application of opicinumab, directed at LINGO-1 in patients with RRMS and secondary progressive MS, was not efficient [40,41].

## 3. Current Disease Modifying Treatment and Symptomatic Therapy for MS

Findings on immune mechanisms from experimental models have opened various avenues for therapeutic interventions. Indeed, substances such as natalizumab or S1P modulators are based on the underlying immunopathology of MS. The number of DMTs for patients with RRMS with varying efficacy and safety is increasing; the most recent advance in treatment is based on B cell inhibition [42]. Currently, there are three CD20-targeted B cell depleting therapies in MS with different safety profiles, such as rituximab, ocrelizumab, and ofatumumab. Ocrelizumab was also approved for the treatment of primary progressive MS [43]. However, though most of the substances are effective in patients with RRMS, efficient substances for patients with primary or secondary progressive disease course are still lacking. Key factors for these substances are efficacy for relapses and disease progression, safety, tolerability, and the convenience of treatment.

Here, we quickly summarize the evolution of DMTs for MS. Interferons beta-1a/b are modulators of inflammatory mediators. Interferon beta-1b was the first interferon approved for treatment of MS in 1993 [44], followed by two interferon beta-1a preparations [45,46]. Several trials showed a reduction in annualized relapse rates of 30–40% compared with placebo and an innocuous safety profile. Glatiramer acetate, which is considered to induce a shift towards a neuroprotective immune response [47], reduces annual relapses by 30% [48]. Dimethyl fumarate (DMF) causes a reduction in annual relapses of 45 and 53%, respectively [49,50]. Its mechanisms of action include effects on nuclear factor erythroid 2-related factor 2, and an increase in oxidative responses in myeloid cells downregulating activated lymphocytes [51,52]. Flushing, abdominal pain, diarrhea, and nausea are common, and few cases of progressive multifocal leukoencephalopathy (PML) cases were reported, often associated with a lymphopenia. DMF treated patients had higher relapse-free survival times compared to teriflunomide [53]. Further, RRMS patients treated with teriflunomide and DMF have similar discontinuation rates [54]. Immune cell migration inhibitors can be separated into non-selective and selective S1P modulators. Fingolimod, a non-selective modulator of the S1P receptors 1, 3, 4, and 5, is a small molecule interrupting S1P signaling, thereby inhibiting egress of immune cells from lymph nodes [55]. Fingolimod showed a 48% and 60% reduction in annual relapses, respectively [56,57]. The safety profile of fingolimod includes effects on heart conduction, elevated liver enzymes, and increased risks of macular oedema and infections, such as herpes viruses, and rare opportunistic infections such as PML [57]. Second-generation S1P modulators are more selective. For example, Ozanimod, which has a weaker effect on the type 3 receptor subtype, and caused a 39% and 49% reduction in annual relapses, compared to interferon beta-1a [58]. No clinically significant bradycardia or second- or third-degree atrioventricular blocks were reported with ozanimod. Further, ponesimod, another selective S1P receptor 1 modulator, revealed a 30% reduction in annual relapses compared to teriflunomide [59]. The monoclonal antibody natalizumab, an adhesion molecule blocker, binds at the a4-integrin of the very late antigen-4 (VLA-4) protein, blocking the migration of immune cells over the blood-brain barrier. Natalizumab reduces the number of annual relapses by 69% [60], however, it is associated with a risk of PML restricting its usefulness. The CD20 monoclonal antibody ocrelizumab was the first DMT approved for PPMS. In patients with RRMS, ocrelizumab reduces annual relapses by 45%, compared with Interferon beta-1a [61]. In patients with PPMS, ocrelizumab reduces the proportion of patients with disability progression by 24% [62]. Rituximab, another CD20 monoclonal antibody approved for rheumatic and hematological conditions, was tested in RRMS [63] and PPMS [64,65]. Notably, rituximab is used in the treatment of several demyelinating disorders of the central nervous system such as MS or neuromyelitis optica spectrum disorders [66]. The study in PPMS showed a similar reduction in progression as ocrelizumab. Application of ofatumumab, which recognizes another epitope on CD20, reduces the number of annual relapses by 50% and 60%, respectively, compared with teriflunomide [67]. In addition, monoclonal antibodies can block proteins inhibiting axon growth and myelination. These antibodies include opicinumab, directed at the LINGO-1, and an antisemaphorin 4D antibody called VX15/2503 [68]. Opicinumab was not efficient in the treatment of MS [41,69].

Despite the frequency of neurologic symptoms such as fatigue or spasticity, the number of symptomatic treatments is limited. The burden caused by symptoms on a daily basis may significantly reduce the quality of life of patients. For example, interferon beta treatment in RRMS may worsen headaches in patients with pre-existing headaches or may cause the appearance of a new headache [70]. Many substances are insufficiently effective against spasticity, tremor, neuropathic pain, fatigue, bladder dysfunction, and sexual dysfunction. Most drugs are prescribed off-label, and well-conducted clinical trials are hardly available. Substances approved for MS include fampridine for gait difficulties, botulinum-toxin for neurogenic bladder dysfunction, and a synthetic cannabinoid for spasticity. There are no effective drugs available for cognitive impairment, fatigue, or severe tremor, which can be incapacitating symptoms. Cognitive impairment is present in 40–70% of patients with progressive MS. The most frequently impaired domains, such as sustained attention, information processing speed, memory, and executive functions, designing and administering rehabilitation training, are needed [71]. DMTs, such as beta-interferons and glatiramer acetate, were suggested to slow the progression of cognitive dysfunction [72].

The development of symptoms originating from axonal degeneration in MS may be prevented by protecting myelin degeneration. Inducing endogenous repair mechanisms in the CNS was a scientific goal over many years. Given the potential of substances such as opicinumab or an anti semaphorin 4D antibody to modulate myelination, this could be a promising strategy to protect axons [73,74]. As mentioned above, the steps of activation, migration, and proliferation of OPCs are required to induce the process of remyelination. Recent insights into the biological mechanisms of cell homeostasis and mitochondrial damage [75], glial-neuronal interactions [76,77], and certain cellular pathways deepened our understanding of the CNS microenvironment and may have opened avenues for therapeutic interventions. Growth factors are an increasingly interesting target for new treatment strategies. Data suggest that glatiramer acetate and fingolimod induce the release of brain derived neurotrophic factor (BDNF) [78]. A connection to FGFs can be established as expression of BDNF is upregulated after knockout of FGFR1 (see paragraph 7). Pharmacological inhibition of FGF(R)s may affect both oligodendrocytes and immune cells, leading to both reduced inflammation and increased remyelination capabilities. Due to the multifarious effects of FGFs on many cells, modulation of this pathway through the receptors promises to be auspicious in the context of MS for the protection of myelin and axons. 

## 4. FGF/FGFR Signalling Controls Myelination in the Adult Mouse CNS

As the FGF/FGFR pathway plays a central role in the cell cycle of oligodendrocytes and their progenitors, it could be a target to enhance remyelination. The FGF family consists of secreted proteins signaling to receptor tyrosine kinases and intracellular non-signaling proteins. FGFs are important early in embryonic development; in adulthood, they regulate cellular processes such as proliferation, survival, migration, differentiation, and metabolism [79]. Activated FGFR phosphorylate proteins of intracellular signaling pathways such as mitogen-activated protein kinase/extracellular signal-regulated kinases (MAPK/ERK), protein kinase B (PKB/Akt), signal transducer and activator of transcription (STAT) and phospholipase C (PLC)γ pathways [79]. Downstream of RAS and PI3K, FGFR signaling regulates several MAP kinases such as ERK1/2, JNK and p38.

The FGF family is one of the most diverse growth factors, and 22 FGF ligands were identified in mice and human [80]. FGFs act in an autocrine or paracrine fashion by interacting with high affinity and different degrees of specificity with FGF receptors present at the cell surface [79]. FGFs are divided into six subfamilies, five paracrine subfamilies, which contain the FGF1 subfamily (FGF1 and FGF2), the FGF4 subfamily (FGF4, FGF5, and FGF6), the FGF7 subfamily (FGF3, FGF7, FGF10, and FGF22), the FGF8 subfamily (FGF8, FGF17, and FGF18), and the FGF9 subfamily (FGF9, FGF16, and FGF20). The endocrine FGF19 subfamily consists of FGF19, FGF21, and FGF23 [80]. Upon binding of FGF to FGFR, a ligand dependent dimerization and transphosphorylation of FGF receptor mediate the downstream signaling, as mentioned earlier [81].

FGFRs are ubiquitously expressed throughout the CNS on presumably all cell types. In oligodendroglial lineage cells, FGFR 1-3 are differentially expressed by progenitors and oligodendrocytes. Specifically, FGFR1 is expressed in all oligodendrocytes; FGFR2 is expressed in mature oligodendrocytes; FGFR3 is expressed in early and late progenitors. In addition, FGFR1 is up-regulated and FGFR2 downregulated by FGF-2 in oligodendrocytes [82]. Further, FGFR signaling is important for the control of proliferation, migration, survival, and differentiation of neurons [80]. FGFRs are required in the presynaptic neuron to respond to FGF22 [83].10 FGFs and 4 FGFR are expressed in the developing mouse CNS. Furthermore, splice variants and crosstalk between ERK and Akt through mammalian target of rapamycin (mTOR) [84] presumably affect the signaling axis, adding more complexity to intracellular FGFR dependent signaling. During brain development, FGF signaling is required for the initial generation of OPC, but not for the proliferation of OPC [85]. In adult mice, myelin growth is controlled by FGF signaling. Oligodendrocyte-specific deletion of FGFR1/FGFR2 decreases myelination associated with a reduction in ERK1/2-MAPK signaling [86]. In adult mice, FGF2-mediated FGFR signaling promotes oligodendrocyte proliferation but inhibits their differentiation and the expression of myelin proteins. Moreover, FGF2 induces demyelination when injected into a ventricle in the CNS of healthy rats [87]. The effects of FGFs on oligodendrocytes were investigated in physiological states and in MS, in vivo and in vitro, to establish that FGF2 and 9 inhibit myelination [87,88,89]. Inter alia, Myelin Regulatory Factor (Myrf)—thought to be an essential component of the process of myelination in oligodendrocytes [90] —is induced by the absence of FGFR dependent signaling [82]. Overall, FGF presumably exert a pivotal role on the remyelination cell program. In the current state of knowledge, FGF1, 2, and 9 activate FGFR1, 2, and 3, which in turn regulate various downstream signals. Taken together, data from the adult CNS of mice point towards a negative role of FGF signaling with respect to myelin generation in oligodendrocytes. 

## 5. FGF/FGFR1 Signalling Pathways Regulate Myelination and Inflammation in MS

**Figure 1:**
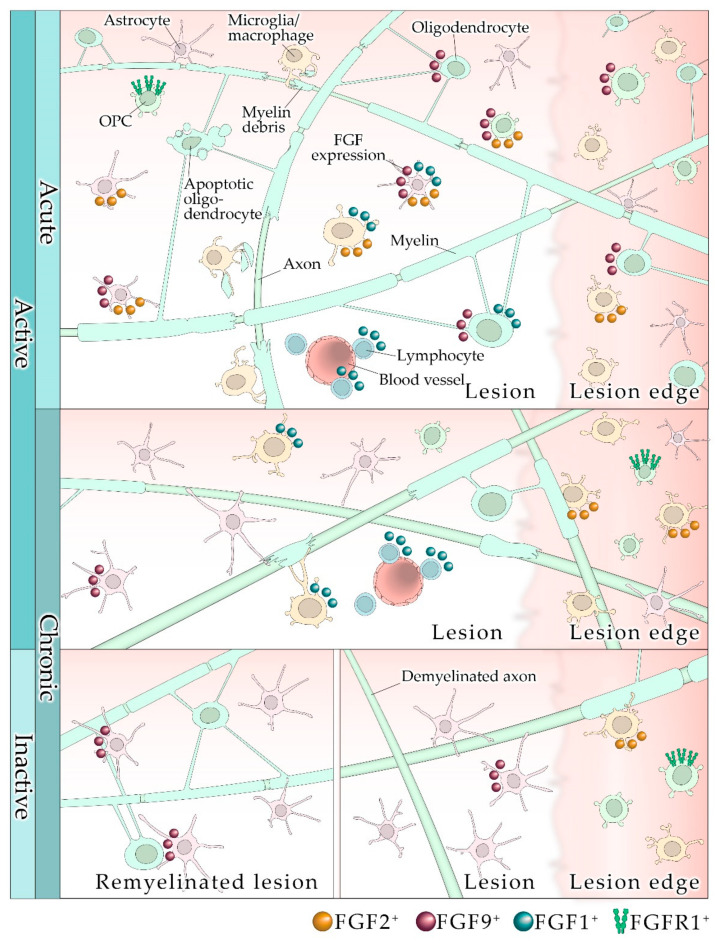
Lesion types and FGF/FGFR expression in multiple sclerosis. Depending on the lesion type, FGFs are associated with myelin damage and inflammation to a varying degree. Abundancy is especially high in acutely demyelinating lesions.

Data on the role of FGF/FGFR in the pathology of MS are limited, possibly due to constraints such as analysis of suitable post-mortem tissue from patients with progressive MS, and the general scarcity of CNS tissue from patients with MS. In post-mortem and in brain tissue from patients with progressive MS, FGF2 is expressed stronger than in controls, however, in this study no correlations were found between FGF2 expression and parameters such as age, disease duration, or the total number of MS lesions [91,92]. In active lesions, an elevated number of FGF1/2^+^ macrophages and astrocytes was observed within and around lesions [91,92], and FGFR1 was upregulated in an OPC subpopulation in active lesions and around chronic lesions. The upregulation of FGF2 by activated microglia/macrophages suggests a role for FGF2 in attracting OPCs and possibly regeneration of myelin [91]. These conclusions come from cell and tissue culture experiments; it remains to be shown whether they pertain to the microenvironment of the CNS. Further, since FGFR1^+^ OPCs were distributed analogously to FGF^+^ immune cells, a positive feedback mechanism by which FGFR1 is upregulated by FGF2 could be operating [93]. In a more recent study, investigation of tissue from patients with progressive MS revealed that FGF2 expression is most prominent in demyelinated lesions [92]. Lesions contain a large number of FGF2^+^ astrocytes; occasionally FGF2 is expressed by OPCs [92]. FGF2 expression correlated positively with CD68^+^ cellular inflammation and negatively with myelination (see Figure 1). FGF2 is known to be functionally pleiotropic. It is possible that FGF2 initially plays a positive role for intrinsic repair, contributing to oligodendrocyte recruitment and proliferation, but later inhibits the differentiation of oligodendrocytes. FGF2 may contribute to a proinflammatory microenvironment, in which neuroprotective autoimmunity prevails (for an introduction see [94]), but when exuberating, it causes damage. In another study, where tissue from patients with secondary progressive MS was analyzed, FGF1 was investigated [95]. The authors showed that FGF1 is prominent especially in remyelinated areas but also—to a lesser extent—in demyelinated lesions, and concluded from tissue culture experiments that FGF1 is a promotor of remyelination. FGF1 is localized in astrocytes, oligodendrocytes, microglia/macrophages, and lymphocytes, and it correlates with both re- and demyelination; it does seem to have an ambivalent role, including negative effects on remyelination through FGFR signaling (see Figure 1). Analysis of FGF2 levels in the cerebrospinal fluid (CSF) of patients with RRMS revealed that concentrations of growth factors positively correlate with disease activity, and high levels of FGF2 were found in relapse [96]. A study of the CSF from MS patients by others [97] showed a trend of elevated FGF2 levels in relapses. The overall abundance of FGF2 in the CSF was very low and was not associated with the ongoing inflammatory progress. Thus, FGF2 levels in the CSF may not adequately reflect MS progression. Others have focused on investigating FGF9, which is expressed by neurons and glia [88,96]. FGF9, expressed by oligodendrocytes and astrocytes, is increased in active lesions and at the edge of chronic inactive lesions. However, FGF9 is almost absent in chronic inactive lesions [88]. The lack of FGF9 in lesions may result in a failure of remyelination and may contribute to the detrimental microenvironment of non-remyelinating lesions. Again, FGF9 has ambivalent roles in tissue and cell cultures. Monocultured oligodendrocytes express higher levels of myelin proteins. This effect of FGF9 is reversed, however, when added to tissue cultures [88]. This underlines the importance and complexity of the CNS microenvironment, where a multitude of growth factors, chemokines, and cytokines will influence cell reactions to certain ligands. This especially applies to the inflammatory CNS microenvironment. Overall, these data suggest that prolonged high glial expression of FGF9 may cause a failure of remyelination. The known distribution of FGF/FGFR in different MS lesions states, which is associated with myelin damage, cytokine expression, and inflammation, and manifests particularly abundantly in acutely demyelinating lesions; this is summarized in Figure 1.

## 6. FGF2 May Be Neuroprotective in EAE

Experimental MS research in recent decades largely relied on animal models such as experimental autoimmune encephalomyelitis [98]. Several MS mouse models were generated to study inflammation, demyelination, and remyelination in the CNS. None of the experimental models covers the entire spectrum of the clinical, pathological, or immunological features of MS. However, the most frequently used sensitization model for MS is immunization of mice with MOG_35–55_. Typical EAE symptoms include paralysis of limbs and gait ataxia [99]. EAE models have proven useful to identify effective therapies for MS [100,101]. Studies on the role of FGF2 in MOG_35–55_-induced EAE suggested beneficial effects of FGF2 on the disease course, inflammation, and neurodegeneration. For example, one-time intrathecal injection of a herpes simplex virus type 1 engineered with a FGF2 gene ameliorated the disease course starting five days after injection to the end of the study [102]. In these treatment experiments, FGF2 appears to be functionally complex, as repeated injections seem to cause reversed effects. However, mice treated with the FGF2 gene induction showed less T cells and macrophages in the spinal cord. Analysis 60 days after the onset of EAE revealed a reduction in myelin damage and axon loss, and OPCs were more abundant in demyelinated areas. The authors suggest that gene therapy with neurotrophic growth factors may be an option to promote recruitment of myelinating cells into demyelinated areas and to avoid side effects caused by systemic application. In agreement with the beneficial findings from the gene therapy study, FGF2^−/−^ mice revealed deleterious effects of FGF2 in EAE [103]. Approximately 40 days after EAE induction, FGF2^-/-^ mice showed a more severe disease course lasting to the end of the study. Analysis of spinal cord tissue did not reveal differences in inflammation, however, at the peak of EAE more CD8+ cells and macrophages/microglia were found in FGF2^−/−^ mice. This finding deserves attention, since high CD8+ cell counts in demyelinating plaques are characteristic for MS. The number of remyelinated fibers and axons was decreased in FGF2^−/−^ mice. Since the clinical differences between FGF2^−/−^ mice and controls were present in the chronic phase of EAE, the authors hypothesized that FGF2 exerts a neuroprotective effect by promoting remyelination in late EAE disease phases. Rottlaender et al. used systemic and not cell-specific genetic ablation implying that the effects of FGF2 may have been caused by a number of cells such as glial cells and immune cells in the periphery and the CNS [103]. Action of FGF2 is versatile, given that repeated induction of high FGF2 levels in the CSF reversed beneficial effects; FGF2 levels expressed by macrophages/microglia increase with the progression of demyelination in EAE [104]. Similarly, in other models such as murine hepatitis virus strain A-59 (MHV-A59), highest levels of FGF2 mRNA coincided with the beginning of remyelination and remained elevated after clinical remission [105]. Here, FGF2 was mainly found in reactive astrocytes. Thus, FGF2 may be part of the initial tissue repair by activating macrophages/microglia and astrocytes which in turn might amplify remyelination. A possible candidate to stimulate FGF2 expression in this model might be CNTF, which was shown to promote neuron survival indirectly after tissue injury [106,107]. 

## 7. FGFR1 and FGFR2 in Oligodendrocytes Increase Myelin and Axon Degeneration and Inflammation in EAE

**Figure 2:**
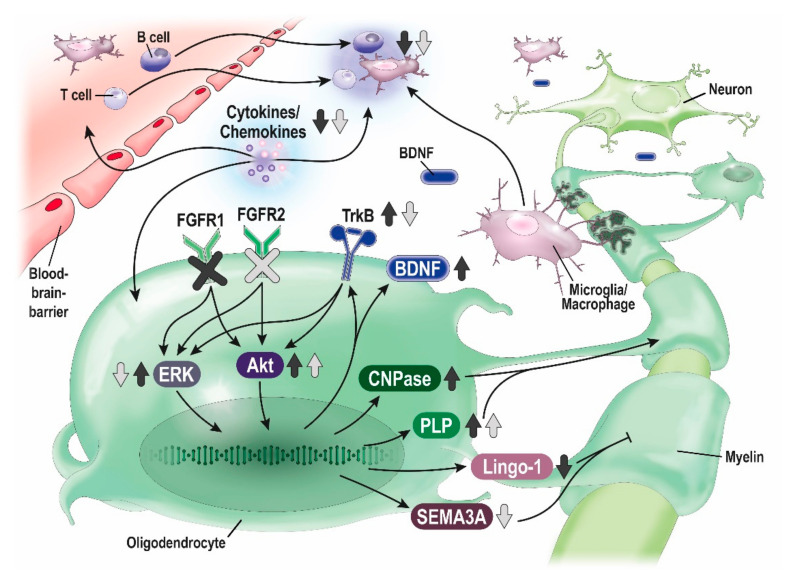
Effects of oligodendrocyte-specific deletion of FGFR1 and FGFR2 in EAE. Proposed neuroprotective and anti-inflammatory mechanisms of oligodendrocyte-specific deletion of FGFR1 (dark grey arrows) and FGFR2 (bright grey arrows) in MOG_35-55_-induced EAE (according to [108] and [109]).

To characterize the role of FGFRs in oligodendrocytes in vivo, researchers developed induced oligodendrocyte-specific deletion of FGFR to eliminate the effects of FGFR signaling through other cell types. In MOG_35–55_-induced EAE, oligodendrocyte-specific deletion of FGFR1^ind−/−^ [108] and FGFR2^ind−/−^ [109] showed a less severe disease course in the chronic phase of EAE. There, myelin and axon degeneration, and the number of T cells, B cells and microglia/macrophages was reduced in the spinal cord in FGFR1^ind−/−^ [108] and FGFR2^ind−/−^ mice [109]. In FGFR1^ind−/−^ mice. An increase of ERK/Akt phosphorylation and increased expression of the BDNF and its receptor TrkB were observed, further LINGO-1, an inhibitor of remyelination, was downregulated [108]. These findings suggest that oligodendrocytes can elicit an intrinsic regulatory response upregulating the BDNF/TrkB pathway to facilitate remyelination. FGFR2^ind−/−^ mice, while showing a similar disease course, presented differently on the molecular level: downregulation of ERK and upregulation of Akt phosphorylation were found. FGFR2^ind−/−^ mice showed a downregulation of the remyelination inhibitors SEMA3A and TGF-beta, and FGF2 [109]. Since only Akt was raised in the chronic phase of EAE, other pro-myelinating mediators than ERK or BDNF/TrkB may account for decreased myelin degeneration. Data from these EAE studies on the function of FGFR in oligodendrocytes suggest that FGFR in these cells mediate inflammation and neurodegeneration (see Figure 2).

Another disease model of MS is based on the treatment with cuprizone, which induces toxic demyelination without inflammation [110]. In an acute cuprizone model, oligodendrocyte-specific deletion of both FGFR1 and FGFR2 did not affect myelin recovery; in the chronic cuprizone model deletion of both FGFR1 and FGFR2 resulted in less remyelination [111]. However, specific deletion of FGFR1 in the chronic state promoted functional recovery and remyelination [112]. These data suggest that while cell-autonomous FGF signaling is redundant during recovery of acute demyelinated lesions, it facilitates regenerative processes in chronic demyelination. Thus, excessive FGF/FGFR signaling could be an important factor for the disturbed initiation of remyelination in demyelinated lesions. Taken together, conditional FGFR deletion studies in MOG_35–55_-induced EAE provided reasonable evidence that inhibition of FGFR signaling may have beneficial effects on demyelinated lesions and inflammation. Further, considering the relevance of FGF/FGFR signaling in adult neurogenesis and neuroinflammation, modulation of FGF2/FGFR signaling could be a target for therapeutics of neurodegenerative disorders, such as multiple sclerosis [113]. Therefore, selective FGFR inhibition may be a promising approach to enhance remyelination, a goal that is unmet by current treatment strategies.

## 8. Summary

As described in the first part, current MS treatments pledge a strong reduction in annual relapse rates to patients suffering from MS. When it comes to progressive disease states, neurodegeneration, and disability, no effective therapeutic strategy exists. Therefore, innovative approaches are needed, to either halt demyelination and prevent disability or regain functionality by neuronal repair. Hereof, the FGF/FGFR pathway is of interest. Increasing evidence shows that FGF signaling is important for the pathology of MS and its disease model EAE. In MS, FGF2 expression correlates positively with macrophages/microglia and negatively with myelination. In contrast to FGF2, FGF1 has been suggested to promote remyelination in MS. In the EAE disease model, intrathecal gene therapy with FGF2 and systemic deletion of FGF2 indicated a beneficial role of FGF2 in the chronic phase of EAE, though inconsistent findings depending on the exposure to FGF2, and a strong dependence on the microenvironment suggests functional diversity of this signaling cascade in the adult. Yet, both approaches were protective for myelin and axons, and the gene therapy also reduced inflammation in the spinal cord.

Overall, discrepancies were reported regarding the role of FGFs and their receptors depending on the animal models, systemic or specific deletion, inflammatory microenvironment, and disease phases. Especially, the ligand has proven to induce ambiguous effects possibly due to its role as a ubiquitous growth factor. Therefore, targeting the receptors might clarify cellular responses. Two studies specifically addressed the function of FGFR in oligodendrocytes. Conditional deletion of FGFR1 and FGFR2 resulted in several changes in the chronic phase of EAE: a less severe disease course, reduced inflammation, decreased myelin, and axon degeneration. The underlying pathology in these studies were changes in ERK and Akt phosphorylation, and the expression of BDNF and several remyelination inhibitors. This increase in expression of BDNF after knockout of FGFR1 is of particular interest, as it is reported that established MS therapeutics such as glatiramer acetate and fingolimod also upregulate BDNF expression [78], thereby possibly constituting part of their efficacy. Based on these findings, inhibition of FGFR may be a promising approach to modulate inflammation and neuroprotection in EAE and possibly MS.

## Abbreviations

CNS	Central nervous system
CSF	Cerebrospinal fluid
DMF	Dimethyl fumarate
DMT	Disease modifying treatment
EBV	Epstein Barr Virus
EAE	Experimental autoimmune encephalomyelitis
FGF	Fibroblast growth factor
FGFR	Fibroblast growth factor receptor
LINGO-1	Leucine rich repeat and immunoglobin-like domain-containing protein 1
mTOR	Mammalian target of rapamycin
MAPK	Mitogen-activated protein kinase
Myrf	Myelin Regulatory Factor
ERK	Extracellular signal-regulated kinases
MS	Multiple sclerosis
MOG	Myelin oligodendrocyte glycoprotein
OPCs	Oligodendrocyte progenitor cells
PROs	Patient-reported outcomes
PLC	Phospholipase C
PML	Progressive multifocal leukoencephalopathy
PKB/Akt	Protein kinase B
RRMS	Relapsing remitting multiple sclerosis
SEMA3A	Semaphorin 3A
STAT	Signal transducer and activator of transcription
S1P	Sphingosine 1-phosphate
TGF-β	Transforming growth factor beta
VLA-4	Very late antigen-4
